# Similar extrapolation of moving objects' locations for perception and saccades

**DOI:** 10.1167/jov.24.9.7

**Published:** 2024-09-09

**Authors:** Eli Brenner, Jeroen B. J. Smeets

**Affiliations:** 1 Department of Movement Sciences, Vrije Universiteit Amsterdam, Amsterdam, The Netherlands

**Keywords:** saccades, illusion, expectations

Lisi and Cavanagh (2024) presented participants with targets moving in their visual periphery. Texture within these targets moved orthogonally to the target motion, as might happen when a flying ball is spinning. This makes one misjudge the direction in which the target is moving (Shapiro, Lu, Huang, Knight, & Ennis, 2010; Tse & Hsieh, 2006). Lisi and Cavanagh asked the participants to make saccades to the targets and then to indicate the directions in which the targets were moving. The texture's motion influenced the directions that the participants indicated, which corresponded with the directions of their ocular pursuit immediately after the saccades. However, according to Lisi and Cavanagh, the saccades themselves did not end on extrapolations of the target's path in the indicated directions of motion. We argue that this conclusion is incorrect: the saccades did land on such extrapolations of the target's path if the extrapolation is performed more realistically.

The critical difference is that Lisi and Cavanagh implicitly assume that perception is consistent across time, so that changes in the target's perceived position are always in the direction of its perceived motion. We assume that the target's perceived position can change independently of its perceived motion and that only the latter is influenced by the moving texture (Smeets & Brenner, 2023). A clear example of perceived motion that is not accompanied by a corresponding change in perceived position is shown in Movie 1. We have previously argued (Brenner & Smeets, 2022) that misjudging the motion but not the position could account for the pattern of errors that is found when making saccades toward the kind of targets used by Lisi and Cavanagh. Here we examine this more quantitatively, on the basis of their own data.

**Figure 1.**
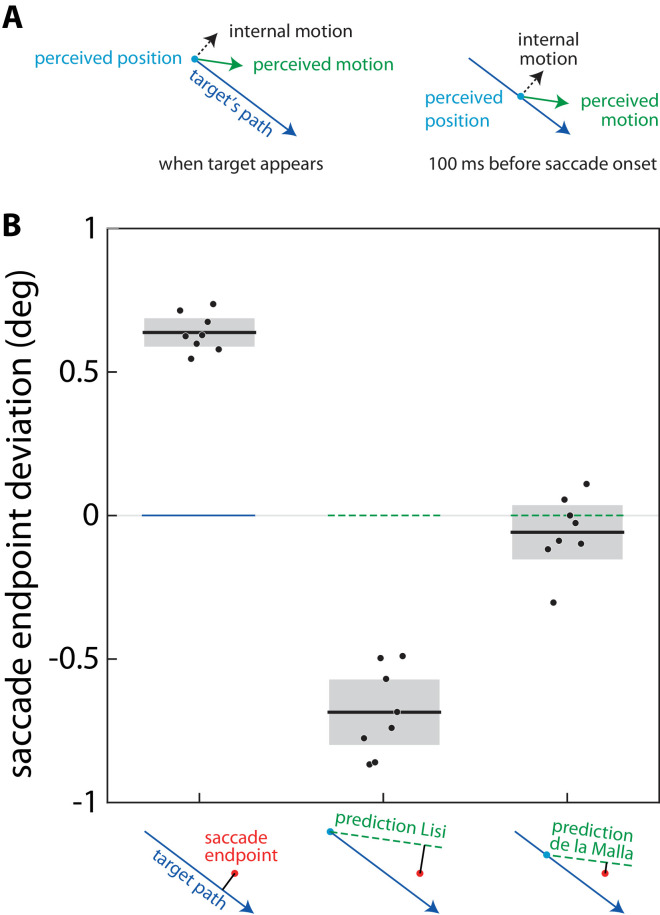
Deviations of saccade endpoints from three possible predictions. (**A**) What we believe participants perceive when the target first appears and 100 ms before saccade onset. The target moves in a certain direction (blue arrow) while the texture moves in a perpendicular direction within the target (internal motion; dashed black arrow) making participants misjudge the target's direction of motion (green arrow). We assume that participants judge the target's position correctly (blue dot). (**B**) The mean distances of saccade endpoints from the target's true path and from two predictions of its path. Dots represent individual participants; lines and shaded areas show means and 95% confidence intervals across participants. The schematic drawings at the bottom illustrate the deviation (black line segment) of a saccade endpoint (red dot) from the path or from the two predictions. Lisi's prediction follows the perceived motion from the moment the target appears. De la Malla's prediction follows the perceived motion from the target's position 100 ms before saccade onset. A deviation is considered to be positive when it is consistent with the misperception of direction (counterclockwise in these schematic drawings). The software used to create this plot from the data provided by Lisi & Cavanagh (2024) can be found at https://osf.io/2eqf3/.

Assuming consistency, Lisi and Cavanagh expected saccades to end along an extrapolation in the perceived direction of motion, starting at the position at which the target appears. They assume that the target's position is estimated when it appears, and this position is combined with the perceived direction of motion to estimate the target's future path. From studies on arm movements, we know that the anticipated movement endpoint is constantly updated on the basis of visual estimates of the target's position about 100 ms earlier (Brenner, de la Malla, & Smeets, 2023). We therefore anticipated that saccade endpoints will fall on an extrapolation based on the target's position about 100 ms before saccade onset (Figure 1A), which is about 120 ms before the end of the saccade. We therefore reanalysed the data of Lisi and Cavanagh (2024) to see whether saccades end along such an extrapolation. We expected this to be the case, because that is how modifying a target's apparent *speed* by moving texture within it influenced how people tapped on it (de la Malla, Smeets, & Brenner, 2018). The influence corresponded with using the incorrectly perceived speed to estimate the target's displacement during the last 100 ms before the tap.

**Movie 1.** Demonstration of motion without a corresponding change in position (based on Flynn & Shapiro, 2018). The diamond on the left appears to constantly move upwards. That on the right appears to constantly move downwards. However they remain more or less aligned. There might be a tiny offset in the percept, as has also been found when texture moves within a target (de la Malla et al., 2018; De Valois & De Valois, 1991), but such an offset is clearly negligible in comparison with what one would expect if the illusory motion were accumulated throughout the time that the illusory motion is perceived.

Figure 1B shows that a prediction based on extrapolating from the target position 100 ms before saccade onset (following de la Malla et al., 2018) is much more consistent with the data than a prediction based on extrapolating from the initial target position (following Lisi & Cavanagh). In their discussion, Lisi and Cavanagh consider that extrapolating the target's motion across about 110 ms for guiding saccades could account for their data, but attribute this to neural pathways responsible for saccades operating on a faster time scale than those responsible for perceptual inferences. Indeed, saccades are probably guided by the best estimate of the direction in which the target is moving at the critical moment for the saccade, whereas reporting a percept after the presentation allows participants to integrate information across the whole presentation. But this does not mean that the extrapolation itself is different, as the authors claim in their title.

According to our interpretation, as well as the one presented in the discussion of Lisi and Cavanagh's article, the saccade endpoint deviations should be independent of the saccade latency. When expressed as directions on the screen (as in Figure 4D of Lisi and Cavanagh's article), this should lead to a slight decrease in magnitude as the saccade latency increases, because the same *saccade endpoint deviation* corresponds with a smaller error in terms of direction on the screen if it occurs later in the movement. No such decrease was found. Possibly, the effect of motion of the texture within the target builds up during the initial part of the target's motion. This deviation from our prediction seems indeed only to be present at the onset of target motion, because saccade endpoint deviations did not depend on the position of the target along its path or on the latency of the saccade for longer durations between the onset of target motion and the saccade (Lisi & Cavanagh, 2015).

Finally, the data suggest that the prediction for the saccade endpoint would be better for a shorter extrapolation time: using the target's position about 10 ms later. It could be that the endpoint is adjusted to some extent during the saccade, so the extrapolation is not based on the position 100 ms before saccade onset, but on the position 100 ms before some other time during the saccade. Moreover, the 100 ms that we used for our predictions is the time it takes for a change in target position to influence the movement of an arm. This includes the time needed to estimate the target's position from the visual information and to activate the relevant muscles. We probably should have considered a shorter extrapolation time, because the latency of responses of the eye might be shorter than those of the hand (Brenner & Smeets, 2015). Considering that the precise delay depends on many factors (Veerman, Brenner, & Smeets, 2008) we would interpret the data of the study by Lisi and Cavanagh (2024) as supporting the notion that moving objects' locations are extrapolated similarly for perception and saccades.
